# Contributions to Physiology

**Published:** 1850-01

**Authors:** 


*Contributions to Physiology.* By BENNET DOWLER, M. D., of New Orleans.

This is the third of the 'Contributions to Physiology,' which the ingenious and able author has communicated to the New Orleans Medical Journal. Of his ingenuity, ample evidence is afforded in his well devised and well executed experiments, and in many of the arguments he brings forward to combat the views of others. Of his ability no less evidence is presented; for although we may be, and are, disposed to question certain of his conclusions, we are ready to admit that they are well and cogently enforced. We would prefer, that the style in which they are frequently put forth was somewhat modified. To our taste it is too turgid, too epigrammatic, and at times too quaint; but this is a matter of taste, on which, as on tastes in general, it would be proverbially idle to dispute.

"The experimental researches now offered to the reader's attention, originated incidentally, during a course of anatomical examinations made upon the great saurian of Louisiana; examinations which have corrected numerous prevalent errors, and which have at the same time resulted in discoveries that are deemed important, if not fundamental, in their bearings upon the doctrines of philosophy; none of which, however, are essential to the purposes of this paper, and, therefore, need not be given now. For anatomical, rather than for physiological reasons, my vivisections have been chiefly confined to the alligator; an animal whose anatomy, physiology, and psychology place it above frogs, turtles, and salamanders, which have been generally relied on by experimenters. How unlike soever the alligator is to man, these latter are more so. If frogs are good, alligators are better. Indeed, Dr. Carpenter, a distinguished physiologist, expressly declares that 'experiments on the nature of this (the nervous) function, are *best* made upon the *cold-blooded* animals; as their general functions are *less disturbed* by the effects of *severe injuries* of the nervous system than those of birds and mammals.'"

The history of the experiments of Dr. Dowler, on "the great saurian of Louisiana" are exceedingly interesting, they would be esteemed miraculous had we not long known the astounding phenomena presented by the amphibia, and by many of the lower classes of animals. It is familiar to every one that the frog will continue sitting apparently unconcerned for hours after it has been eviscerated; that the tortoise will walk about for a long time after decapitation; that the divided portions of a polype will form so many distinct animals; that a common land tortoise, whose brain had been removed, has continued to walk about for nearly six months, groping its way forward for want of vision. The independence of the parts of a wasp, when the head has been separated from the body, are familiar to all: the head will appear to attempt to bite, and the tail to sting. The striking case of the rattlesnake, recorded by Dr. Harlan, is known to every well read *Naturforscher* and physiologist. He cut off the head, and grasping the part of the neck attached to the head with his finger and thumb, the head twisted itself violently, endeavouring to strike him with its fangs. A live rabbit was presented to the head, which immediately plunged its fangs deep into the animal; and when the tail was laid hold of, the headless neck was bent quickly round as if to strike the experimenter.

It need scarcely be said, that the reflecting physiologist, whilst he observes the phenomena in all classes of animals, must exert due caution in his application to man, of laws, which he may consider himself justified in deducing from such phenomena; and caution is especially required when the observations are made on animals like those experimented on by Dr. Dowler. If we place a batrachian under the air-pump, and exclude the air as far as practicable, we observe it apparently but little incommoded; and we know that such animals can exist for years imbedded in rocks; whilst a sudden diminution of atmospheric density to one-half will inevitably destroy all the higher animals. The phenomena, consequently, observed and recorded by Dr. Dowler, are elucidative of the biology of certain beings in the scale of animated creation, whilst they may fail in their application to others, and especially to man; and the same remark is applicable to the conclusions at which he arrives, after detailing his various experiments on the alligator.

"On the whole it may be safely concluded, that voluntary motion is neither directly communicated from, nor regulated by the brain, or the cerebellum; that the muscles, in connection with the spinal marrow, perform voluntary motions for hours after having been severed from the brain; that these motions are not only entirely independent of the brain, but may take place, though imperfectly, after the destruction of the cord itself; that the trunk, as well as the brain, thinks, feels, and wills, or displays psychological phenomena; that the *sensorium* is not restricted to a single point, but is diffused, though unequally, or in a diminished degree, in the periphery of the body; and that actions which take place after decapitation, as described above, are in absolute contrast to *reflex actions*, being sensational, consentaneous, voluntary, and in other respects, dissimilar."—p. 342.

We give in his own words the satisfactory and suggestive detail of his experiments.

"Sept. 8th, 1849.—*Experiments on the Alligator.*—Dr. S. Powell, of this city, witnessed all, and aided in most of these experiments: this account was written the same day, and was read to, and approved by him.

"A longitudinal incision in the neck, from above, was made through the thick mass of muscles upon the cervical vertebræ—the vertebræ and the spinal cord were divided completely; the finger was passed between the divided parts. In about three quarters of an hour after, a traverse incision was made midway between the shoulders and hips—the spine, with the cord, was divided with the saw, the parts were separated so as to admit the finger between the divided ends of the cord, exposing the abdominal cavity. In about half an hour after this second division, the animal was placed on its back, and the whole of the viscera were slowly dissected, and removed from the body. The sympathetic was destroyed. This last dissection occupied about one hour. During the latter part of this process, and some time after the removal of the organs, the animal died, having lived after the first section, about two and a half hours.

"During a period of more than two hours, this animal displayed complete intelligence, volition, and voluntary motion in *all divisions of the body.* It saw, heard, felt, defended itself, showed anger, fear, and even friendly attention to its keeper, a black boy. This latter manifestation, is so extraordinary in an alligator, that I will notice it first, though it was not verified, until after the second division of the spinal cord. This animal, with several others, were presented to me, by my friend Dr. Young, when leaving this city, to visit Europe, during the last summer. Mr. Barbot (at whose apothecary store this animal had been kept, with the others, for many months) informed me, some weeks ago, that it had become fond of the black boy, who had taken care of it. The experiments had lasted more than an hour—the animal had been much exhausted—had lost much blood, and, for a time, scarcely seemed to take notice, when Mr. Barbot proposed to bring up to the third story his servant, its keeper; the boy went near it—he called it in a kind of gibberish style—it raised up its head and turning towards him, gently opened its mouth—looked quietly at him without its usual menace. All four of the gentlemen then present, agreed that it recognized the boy, and that it manifested affection for him. The boy repeated his fondling calls (gibberish) several times, and with the same results. It saw him, followed him with its eyes, and knew his voice. Its mute language could not be mistaken. It was the first time I had seen affection of this kind in alligators. I have kept some nearly a year. They always feared, or menaced me, though I always fed them. For nearly two hours, the animal watched our operations: on approaching too near it, so as to excite its fears, it raised its head, opened its mouth to bite, directing its head to the right or left, to attack its enemy. It threw the nictating membrane over the eye, on perceiving a body approaching near the cornea, in order to defend the same; and this, too, in advance of actual contact. It retained the sense of hearing: for on making a noise by striking a board, without advancing towards the head, it looked angry, and opened its mouth to bite.

"In the meantime, the other parts of the body (though its spinal marrow was divided in two places,—in one severing most of the muscles of the back) manifested sensation, volition, and combined or complex motions of a vigorous character. This was not all. For, the lateral muscles of the body not divided, together with the hind legs, were adapted so as to aid the forelegs in removing fire, or a pricking body, *above the part divided.* In fact, the forelegs, and hindlegs, mutually aided each other, notwithstanding the *intermediate division of the cord.*

"After nearly two hours spent in this kind of experiment, it was found on dissecting the viscera, and sympathetic, the animal lying on its back, that it directed its limbs to the place where the knife was applied in dissection.

"The heart continued to act as long as it was observed, even after having been roughly handled, emptied, and removed from the body.

"For want of suitable instruments to divide the spine, the vertebræ were injured, and the muscles were extensively divided, which of course, diminished the brilliancy of the results.

"October 30th, 1847.—*Experiments on an Alligator, nearly three feet long;* by Dr. Young, Mr. Barbot, and myself:—Five grains of strychnine were dissolved—half of which was thrown into the gullet —a small portion was regurgitated; in twenty-five minutes several convulsive contractions took place in the general muscular system.—The residue of the mixture was now given;—a portion was again rejected—the convulsive contractions increased—tetanic rigidity followed—the jaws forcibly closed—the limbs became stiff and straight. In one hour after taking the first portion of strychnine, the animal appeared nearly dead—there was only an occasional motion on the hind legs, tail, and in the nictating membrane. Dr. Young now began to dissect the animal, with the view of preserving its head, and skin. The limbs had ceased to be rigid, and were sometimes directed intelligentially. The spinal cord was served in the neck,—a probe passed down the spinal canal two or three inches below the part giving off the axillary plexus, breaking up the texture of the cord completely; one spasmodic jerk followed in the forelegs, on introducing the probe. For an hour after destroying the cord, the forelegs contracted, though far less frequently, less forcibly, and less definitely, than the hind legs and tail. The heart for three hours, that is, as long as observed, acted regularly, both before and after its removal from the body.

"April 13th, 1848.—At 10 A. M., I observed an alligator, *in articulo mortis*, (its health had been declining for some days, owing to an inflammation of the bowels with ulceration, as the post mortem examination showed;) it was unable to walk, but watched my movements. In fifteen minutes afterwards, it appeared to be quite dead. It was placed on its back for dissection; it moved its limbs intelligentially for several minutes. The dissection lasted seven hours; during nearly half of this time, the heart or ventricles continued to beat about fourteen times per minute—the right auricle, about twice as often. The heart was separated from all its annexiæ, but did not cease to act, until after its ventricles had been roughly probed. Thus freed from blood, and removed from its connections with the nerves, it acted with regularity, as before.

"May 12th, 1848.—Having observed that an alligator had become feeble, I determined to kill it for dissection. On taking hold of it, it seemed much alarmed, and cried several times, Houpe! Houpe! This is the only articulate sound that I have ever heard from an alligator, and it is, I believe, peculiar to the young animal, and is never uttered but when danger is suspected; it appears to be the synonym of the word *Help*, the sound of which, it very much resembles. It hissed, and attempted to bite. The upper portion of the skull, including a horizontal stratum of brain, was removed. Hæmorrhage, to a considerable extent, followed; the eyes closed. The animal no longer attempted to bite. It performed, however, a series of voluntary motions, intelligently directed, to ward off injuries. The entire brain and the medulla oblongata were removed, without diminishing its power to direct its limbs to any part that was pained by the slightest touch of a pin or knife. A metallic rod was passed many times within the spinal canal, completely destroying the spinal marrow beyond the hips. The animal appeared to die very soon, the tail excepted. It was, however, afterwards found, that both voluntary motion, and sensation, remained, though their manifestations were greatly impaired. The forelegs were slowly and feebly directed towards irritated parts; these motions disappeared in a very few minutes. The tail twitched frequently, for an hour after, as if pained by the dissection of the trunk, and viscera. Both before and after its removal from the body, the heart acted regularly for four hours. The right auricle was the first to collapse.

"May 22d, 1848.—Dr. Young, and myself, (aided by Mr. Barbot,) performed the following experiments, upon a stout alligator, four feet long. Two men drew several strong twines with all their strength around the animal's neck, but this, in ten minutes, did not produce either strangulation, or palsy, though the force of the limbs was diminished. Decapitation was now performed. The great carotid, which threw out blood freely, was tied after three or four ounces had been lost.

"For more than an hour after decollation, sensation, perception, vision, passion, and voluntary motion continued in the head. It saw its enemies—opened its mouth to bite, at the proper time—nictated, when a foreign body approached the eye. The pupils responded, naturally, to the degrees of light.

"The headless trunk for three or four hours, during extensive mutilations by two operations, manifested, in a still higher degree, sensation, intelligence, definite, well directed muscular actions. There was as usual, a complete loss of progressive or forward motion. The tests used to elicit sensation, and voluntary movements, were pinching, puncturing, and burning. Its sensibility and motions appeared to be nearly as acute, quick and varied, as in the unmutilated animal. The direction of the limbs was not such as could be deemed habitual, as in walking and swimming. Some of these motions are of difficult execution in the entire animal, from its anatomical conformation; such as reaching up between the shoulders or hips, to remove an irritant. All of the muscles that could in anywise contribute to the will and aims of the animal, as in curving the body or tail, so as to bring the irritated part within the range of the appropriate limb, etc. There was not a single unmeaning or convulsive motion. These motions, altogether volitional, began, continued, and ended with the pain producing cause. For hours, during the dissection of the viscera, the limbs, when unconfined, were directed in the intelligential manner. While operating on the organs connected with the sympathetic or ganglionic system, these motions were less vigorous and less frequent, than those noticed during operations on the periphery. It was necessary to tie the animal on its back, during the dissection, to restrain its intelligential motions.

"The heart remained *in situ*, nearly four hours; in the meantime, all its annexing vessels had been severed—its associated organs removed, without destroying its pulsatory action, which, in four hours, declined from 36 to 16 per minute. It was roughly handled; its blood emptied out; it underwent pressure and considerable desiccation, but was still active when the observations ended. On other occasions, I have observed the continuous action of this organ, for seven hours, proving that the favorite assumption of physiologists concerning the blood, as being the necessary stimulus to the heart's motion, is an error. There is a simpler explanation, namely, the inherent force of that hollow muscle itself.

"June 5th, 1848.—Dr. Young, Mr. Barbot, and myself, performed the following experiments on an alligator three feet and seven inches in length, occupying a period of seven hours, during the greater portion of which, these gentlemen were constantly present. The animal was not very vigorous. It had a congenital mal-formation or deficiency,—or, what is more probable, it had been early in life mutilated, by which the entire tongue, and the whole flooring of the under jaw, were removed. The skeleton of the lower jaw was covered with a thin, white, dense membrane, all the soft parts having been removed. The animal was emaciated, and highly anemic; its blood was thin and pale, owing, doubtlessly, to the difficulty of catching and swallowing food, in the absence of the tongue, and the flooring to the under jaw. The animal was decapitated, and the great artery of the neck was ligated. On touching the lid, the eyes closed. It bit when a stick touched its teeth. On passing a wire into the foramem magnum, and breaking up the brain, these actions ceased. The sensational, intelligential, motory and volitional phenomena of the trunk, were the same as in the cases already described. About one hour after decapitation, a wire was passed down the spinal canal beyond the hind legs. By repeated manipulations, the texture of the cord was completely disorganized. The vigor and promptness of its intelligential motions were greatly impaired, but not wholly lost. During an hour after this destruction of the cord, punctures, or fire, caused slight motions of a definite character, like those before decapitation, but not constantly. The contractions of the heart were 48 per minute. The intestines, seven hours after decapitation, and five after removal from the body, presented, as usual, contractile phenomena, of a peculiar character.

"August 20th, 1849.—*Experiments on two alligators; each about three feet long.* [Circumstances, not necessary to mention, prevented me from taking full notes at the time of these vivisections. Drs. Cartwright, Smith, Nutt, Powell, Hire, and Mr. Barbot, were present, together with several gentlemen not of the profession, among whom was Professor Forshey.] The alligator, No. 1., was tied on its back. The trachea was ligated in the middle of the neck. No blood was lost. The incision was closed with stitches, and strips of adhesive plaster. The animal was returned to its den, where it was found, apparently dead, about half an hour after. I proceeded to dissect the viscera for a few minutes, when at the request of Mr. Forshey, (a learned and able cultivator of science,) the ligature was removed from the windpipe. The latter was opened. A tube was introduced into the opening. The lungs were repeatedly inflated by Mr. Forshey. The animal was, thereby, soon restored to life. I proceeded to demonstrate the viscera, and to remove the organs. After this was done, (which occupied about two hours,) the animal ceased to show any signs of sensation, or voluntary motion. It lived, after the ligation of the trachea, a much shorter time than decapitated alligators. The heart, both before and after its removal from the body, maintained its contractile motions, as long as observed, that is, for three hours. The apparent death from the tying of the trachea, in so short a time, was a result that I did not expect, because, I had often taken what I supposed to be effectual means to ascertain whether these animals breathed, when left undisturbed, but I never could detect them in the act of breathing, though, when alarmed or angry, they hiss and blow almost constantly. Baron Humboldt says, from personal observation, that they live two or three days without respiration at all—*sans respirer de tout.*

"In this experiment, the animal did not appear to suffer but little, immediately, from the ligation of the trachea. Before the removal of the ligature, and the inflation of the lungs, life seemed quite extinct —the limbs relaxed—the body supple and motionless. If my recollection be accurate, the *incipient dissection*, (that is, before pulmonary inflation,) did not elicit any sensational, or volitional phenomena.—If this be so, (and it is worthy of being tested by experiment,) it would seem that this form of death is more complete, than that by decapitation. After the latter operation, however, the removal of the lungs does not interfere with the phenomena, as already narrated.

"*Experiments on the alligator, No.* 2 : (The same gentlemen as before mentioned, were present :) The decollation was not followed by a projecting stream of blood, as is usual; no ligature was applied to the great artery of the neck. The dull hatchet used in severing the spine of the neck, had probably bruised the artery as in torsion and gun-shot wounds. Hence the hæmorrhage was not great, though considerable.

"I carried the handle of the knife towards the eye, to ascertain whether it would wink, whereupon the ferocious, separated head, sprang up from the table with great force, at me, passing very near my breast, which received several drops of blood ; it alighted upon the floor, from six to eight feet distant from its original position ! It missed me, because I was standing at the side, and not in front of the head. Although, I have examined carefully, all the muscles of the head, I cannot find one that accounts for this feat of combative muscular motion. The angles of the mouth recede so much in this animal, that after decollation, including the medulla oblongata, the head seems almost like two separate pieces,—the superior and the inferior maxillary bones, being joined chiefly by the great masseter muscles, for only a short distance. These great muscles, (the masseters), which are curved having their concavity anteriorly, are adapted only to vertical action, as in biting—the great muscles of the tongue act backward and upward against the palatine region :—whence then this quick, violent, forward motion, or rather, as in this case, diagonal leap of six or eight feet—for the head deviated to the left, where I was standing, evidently with the intention of biting me? The trunk, in this, as in all cases, possessed no power of forward motion. This curious fact with respect to decapitated animals, noticed by M. Magendie, and other vivisectors has been attributed to the *loss of the cerebellum;* but whether this loss of forward motion in the alligator, be owing to a division of the spine, and great muscles, or to the separation of the larger or smaller brain, or both, is not very evident, yet the fact which I have noticed respecting the forward motion of the separated head, is, perhaps, a circumstance favorable to this view. That a voluntary, spontaneous and powerful motion,—in fact a diagonal leap, should be performed by the separated head, must therefore appear astonishing to one acquainted with the muscular organization. It is difficult to understand, how the cerebellum could thus act alone.

"For about two hours, the headless trunk of the last mentioned alligator, exhibited such phenomena as are usually attributed to the brain, namely, sensation, volition, and intelligential motion, as tested by the application of bits of ignited paper, wounds and the like, whereupon, the usual indicants of pain were elicited with great promptness and precision: it trembled, receded, rolled over, curved, placed its limbs accurately to the exact spot, and removed the offending cause. In certain places this was exceedingly difficult, as on the spine between or near the shoulders, or hips. It always used the limbs the best adapted for the purpose. If the fire was too remote, as when applied to the tail, the whole body was thrown into the most favorable position, for the purpose of reaching, and removing the same. If the fire was placed on the table, in a position to annoy, yet without touching, the animal, as if endowed with sight, reached, and always accurately, to the exact spot, and either extinguished the fire, or removed it. As upon former occasions, if the animal found that the fire was continued at the same spot, and that it could not remove it, which was sometimes the case, owing to continuous, or repeated applications, and carefully manœuvring, it curved the body—scratched violently, manœuvred skillfully, and then as a last resort, rolled quite over, laterally, always *from,* never *towards* the fire and operator.

"After these experiments had progressed for some time, Dr. Cartwright desired me to cut off the neck close to the shoulders. This was done, but the intelligential, sensational, and volitional motions continued as before—p. 20.

